# The role of nutritional risk in inflammatory bowel disease: a serial mediation model through fecal calprotectin and depression leading to impaired quality of life

**DOI:** 10.3389/fnut.2026.1891598

**Published:** 2026-09-08

**Authors:** Jiamei Shen, Shiqi Xie, Yan Zhang, Ying Xie, Huilin Ye, Jianrong Zhou

**Affiliations:** 1 Department of Nursing, The First Affiliated Hospital of Chongqing Medical University, Chongqing, China; 2 Chongqing Medical University, Chongqing, China

**Keywords:** depression, fecal calprotectin, gut–brain axis, inflammatory bowel diseases, nutrition, quality of life

## Abstract

**Background:**

Patients with inflammatory bowel disease (IBD) frequently face high nutritional risk, persistent intestinal inflammation, and a high prevalence of comorbid depression. Dysregulation of the gut–brain axis is hypothesized to mediate the association between nutritional status and quality of life (QOL). However, the serial mediating roles of fecal calprotectin (FC) and depression in this pathway remain unclear. This study aimed to examine this serial mediation and provide evidence for developing integrated care models centered on nutritional management to modulate inflammation, manage psychological complications, and improve patient outcomes.

**Methods:**

This cross-sectional study enrolled 327 IBD patients from four tertiary hospitals in Southwest China. Nutritional risk, depressive symptoms, and QOL were assessed using the Nutritional Risk Screening 2002 (NRS2002), the Patient Health Questionnaire-9 (PHQ-9), and the Inflammatory Bowel Disease Questionnaire (IBDQ), respectively. FC levels were measured using an enzyme-linked immunosorbent assay (ELISA). The serial mediating effects were examined using Model 6 of the SPSS PROCESS macro with 5,000 bootstrap resamples.

**Results:**

Nutritional risk was positively correlated with FC (*β* = 1.866, *p* < 0.05), which in turn was positively associated with depression (*β* = 0.021, *p* < 0.05). Depression was negatively associated with QOL (*β* = −1.417, *p* < 0.001). The total indirect effect accounted for 43.86% of the total association and consisted of contributions from three pathways. Of these three pathways, the independent mediating effect of depression alone contributed 8.55%, and the serial mediating effect of FC and depression accounted for 6.36%; both were statistically significant. The remaining portion of the total indirect effect was attributable to the non-significant pathway via FC alone.

**Conclusion:**

Nutritional risk is associated with impaired QOL in IBD patients both directly and indirectly through a sequential pathway involving intestinal inflammation (FC) and depression. These findings suggest a shift in clinical practice toward an integrated care model anchored in proactive nutritional management, coupled with concurrent monitoring of inflammation and screening for depression. This model presents a targeted strategy for modulating inflammation, managing comorbidities, and ultimately improving symptoms and QOL in IBD.

## 1 Introduction

Inflammatory bowel disease (IBD), encompassing Crohn’s disease (CD) and ulcerative colitis (UC), is a chronic, recurrent, immune-mediated intestinal disorder (1). In Asia, the prevalence of IBD is increasing rapidly, and the number of affected patients is projected to surpass 4 million by 2035 (2). A major challenge in contemporary IBD care is moving beyond the traditional discipline-limited model that targets only intestinal lesions toward comprehensive, patient-centered care. By implementing multidisciplinary integrated management covering evidence-based therapeutic regimens, mental health assessments and interventions, screening for social determinants of health, and dietary, nutritional, and lifestyle regulation, clinical practice can transition toward value-based healthcare (3). Nutritional risk is a major and prevalent comorbidity of IBD that may emerge even during disease remission or at an early disease stage, with an incidence ranging from 6 to 70% (4). Nutritional risk can weaken pharmacological efficacy, increase the incidence of surgical complications and the rate of hospitalization, prolong hospital stays, and impair patients’ quality of life (QOL) (5). Previous studies have confirmed that severe malnutrition is an independent risk factor for mortality among IBD patients and directly undermines their long-term survival and overall clinical prognosis (6). Accordingly, systematic nutritional management has become a cornerstone of improving clinical outcomes in IBD patients rather than merely a remedial strategy for established health issues.

The biopsychosocial medical model posits that health and illness result from interactions between biological, psychological, and social factors (7). In IBD patients, nutritional risk and intestinal inflammation are closely associated and represent two pivotal biological factors. Nutritional risk can suppress cellular immunity, complement activity and phagocytic function, impair local and systemic immune competence, and increase the risk of infection, thereby potentially triggering or exacerbating intestinal inflammation (8). Conversely, intestinal inflammatory responses require substantial energy and continuously deplete nutrients and immune proteins necessary for anti-inflammatory reactions, ultimately forming a vicious cycle of intestinal inflammation and nutritional risk (4). Notably, fecal calprotectin (FC) serves as a specific biomarker of intestinal inflammation, and its level is significantly and positively correlated with the severity of endoscopic mucosal inflammation and clinical disease activity in IBD patients (9). Accumulating evidence has confirmed that the pathological progression of IBD can affect the brain through the gut–brain axis (10). Up to 35% of IBD patients experience comorbid emotional disorders such as anxiety and depression (10). In the majority of cases, these emotional disorders stem from impaired communication along the gut–brain axis; specifically, intestinal inflammation can induce depression through this axis (11). Unfortunately, comorbid depression in IBD patients can trigger somatization symptoms, lower the threshold for pain tolerance, aggravate the perceived severity of intestinal symptoms, including abdominal pain and diarrhea, and further reduce dietary intake (10, 12). Depression-induced social withdrawal, anhedonia, and negative cognition impair social functioning and treatment adherence, which, in turn, diminish patients’ QOL across multiple dimensions (13, 14).

The majority of previous studies have separately explored the independent single-pathway effects of FC, depression, and nutritional risk on QOL. Few studies have systematically quantified the integrated mechanisms through which nutritional management, as a core clinical intervention, improves symptoms and QOL through two pathways: regulating intestinal inflammation, as reflected by FC, and managing psychological comorbidities, as represented by depression. This gap results in insufficient targeted evidence for integrated inflammation-, psychology-, and nutrition-based management strategies, particularly evidence specific to Asian populations. Understanding these underlying mechanisms is essential for establishing comprehensive strategies centered on nutritional interventions that synergistically control inflammation and depressive symptoms to improve patients’ QOL. Based on this theoretical framework and the existing research gaps, the present study proposes the following hypotheses:

H1: Nutritional risk is significantly and negatively correlated with QOL in IBD patients.

H2: FC has an independent mediating effect on the association between nutritional risk and QOL.

H3: Depression independently mediates the association between nutritional risk and QOL among IBD patients.

H4: FC and depression constitute a serial mediating pathway that jointly influences the effect of nutritional risk on patients’ QOL.

This study aims to explore the sequential relationships between nutritional risk, FC, depression, and QOL in IBD patients based on the biopsychosocial medical model and the gut-brain axis theory. It seeks to provide empirical evidence for constructing an integrated chronic disease management model for IBD that centers on nutritional management and incorporates inflammation control and psychological support. Additionally, this study offers new perspectives on regulating inflammatory status and improving patients’ overall health outcomes through nutritional interventions.

## 2 Methods

### 2.1 Study design

**Figure 1:**
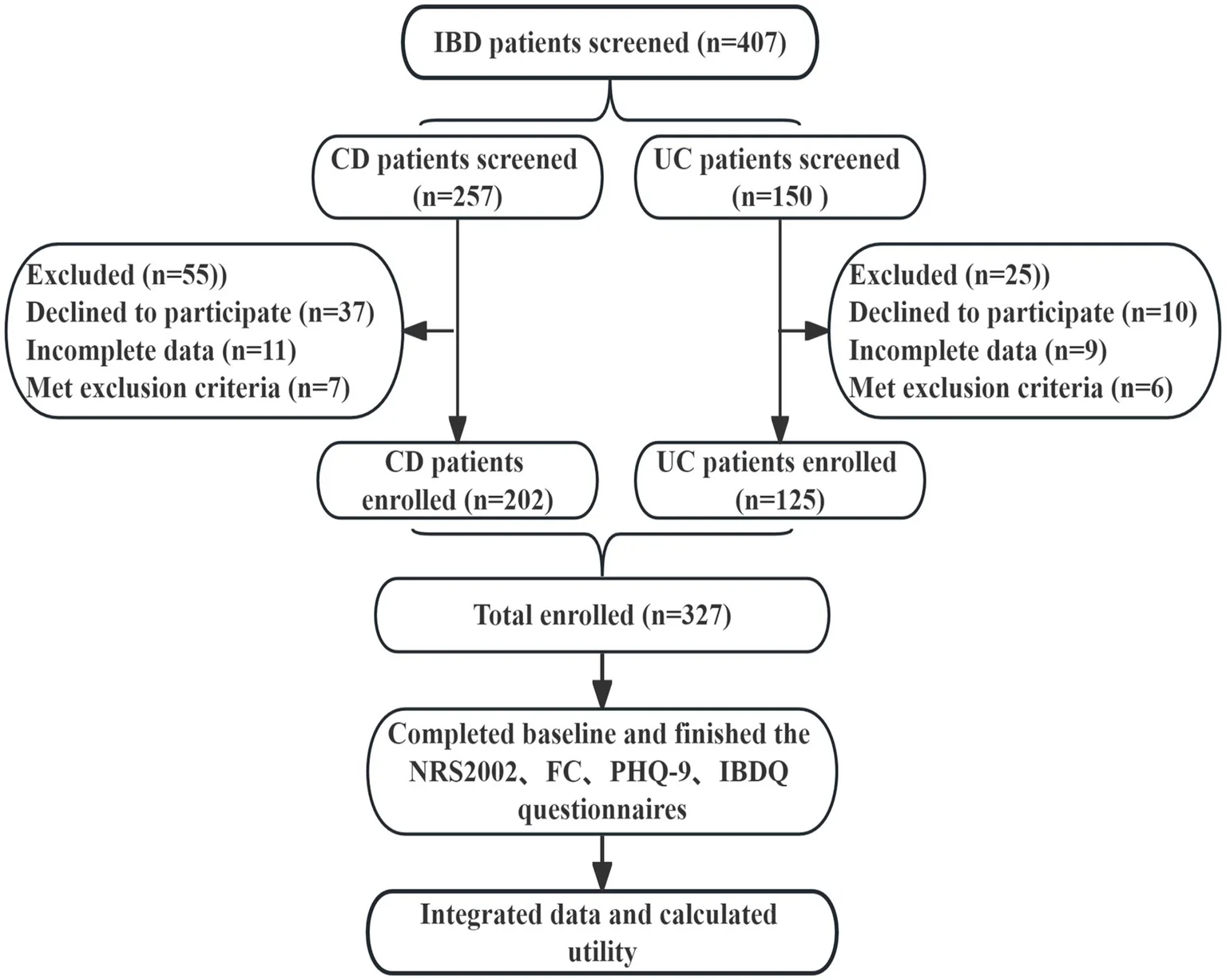
Flowchart of the study.

This multicenter cross-sectional study investigated sociodemographic profiles, nutritional risk, depressive symptoms, and QOL among IBD patients. Convenience sampling was used, and IBD patients admitted to four tertiary Grade A hospitals in Southwest China were enrolled between August and December 2024. In accordance with the methodological principle for mediation analysis, the recommended sample size is generally 10–20 times the number of independent variables. Given that this study included 4 core variables and 12 covariates, the minimum required sample size was 160 (15). The sample size was further calculated using PASS 15.0 software. Based on previous serial mediation studies in IBD, the effect size was derived from a pilot analysis of 50 patients from our cohort, which yielded a serial mediation effect of approximately 0.12–0.18, and from prior literature reporting similar mediation effects in IBD populations. The serial mediation effect size was conservatively set at 0.15 (a moderate effect size for social and clinical mediation analyses) (16, 17), with *α* = 0.05 and a statistical power of 80%; thus, the calculated minimum sample size was 273. Considering a 20% rate of non-response, incomplete questionnaires, or missing data, 327 participants were finally enrolled, satisfying all statistical requirements (Figure 1). All selected hospitals have established specialized IBD centers and use standardized multidisciplinary treatment models involving gastroenterologists, specialist nurses, and clinical dietitians, enabling them to deliver systematic health education and standardized follow-up management.

#### 2.1.1 Inclusion criteria

The inclusion criteria were as follows: ① a confirmed clinical diagnosis of CD or UC; ② a disease duration of ≥6 months; ③ age ≥18 years; ④ voluntarily provided written informed consent; and ⑤ intact cognitive function.

#### 2.1.2 Exclusion criteria

The exclusion criteria were as follows: ① preexisting mental disorders or cognitive dysfunction; ② comorbid malignant tumors or end-stage failure of major organs (including the heart, liver, kidneys, and lungs); and ③ severe acute IBD exacerbation requiring emergency surgery.

Fecal samples for FC measurement were collected within 24 h of questionnaire administration. All investigators received standardized training in questionnaire administration and anthropometric measurements before study initiation. The response rate among eligible patients approached for enrollment was 80.3%. On-site laboratory researchers supervised the entire specimen quality-control procedure.

### 2.2 Clinical and sociodemographic characteristics

This questionnaire was developed by the research team and covered clinical and sociodemographic data. Sociodemographic variables included gender, age, place of residence, education level, marital status, employment status, and household income. Clinical indicators included disease duration, body mass index (BMI), surgical history, and disease type. For disease severity evaluation (18), the Crohn’s Disease Activity Index (CDAI) was used for CD patients. Specifically, a score of ≤150 indicated clinical remission, 151–220 indicated mild disease activity, 221–450 indicated moderate disease activity, and >450 indicated severe disease activity. For UC patients, the modified Truelove and Witts grading criteria were used to classify disease status as remission or active disease. The active stage was further classified as mild, moderate, or severe according to defecation frequency, hematochezia severity, systemic manifestations, and laboratory indicators. The Global Leadership Initiative on Malnutrition (GLIM) criteria were used as the reference standard for detecting malnutrition in ulcerative colitis (UC).

### 2.3 Fecal calprotectin

FC was measured using an enzyme-linked immunosorbent assay (ELISA). The corresponding detection kits were purchased from Guangzhou Fengrun Biotechnology Co., Ltd. The unit of measurement was μg/g, and the reference value was <50 μg/g. All specimens were processed uniformly by dedicated personnel and tested in duplicate wells. The intra-assay and inter-assay coefficients of variation were both <5%, and strict quality control was maintained throughout the experiment.

### 2.4 Nutritional risk assessment

The Nutritional Risk Screening 2002 (NRS2002) was developed by the European Society for Clinical Nutrition and Metabolism (ESPEN) in 2002 (19). It was used for the initial screening of nutritional risk in hospitalized patients and comprises three components: impaired nutritional status, disease severity, and age scores. The maximum total score was 7 points. Participants with a total score of ≥3 were considered to have nutritional risk, whereas those with a score of <3 were considered to have no nutritional risk. The applicability of NRS2002 has been validated in Chinese IBD patients, with a Cronbach’s *α* of 0.853 (20). In this study, the Cronbach’s *α* for NRS2002 was 0.876.

### 2.5 Depression

The Patient Health Questionnaire-9 (PHQ-9) was used to screen patients for depressive symptoms (21). This unidimensional scale consists of nine items that assess core depressive symptoms. All items are scored on a 0–3 Likert scale, and a total score of >4 indicated the presence of depressive symptoms. The Chinese version of the PHQ-9 has been widely used and has shown good reliability and validity among IBD patients (Cronbach’s *α* = 0.887) (22). In this study, the Cronbach’s *α* for the PHQ-9 was 0.819.

### 2.6 Quality of life

The Inflammatory Bowel Disease Questionnaire (IBDQ) was originally developed by Guyatt et al. (23). It contains 32 items across four domains: bowel symptoms, systemic symptoms, emotional functioning, and social functioning. Each item was scored on a 7-point Likert scale, with scores ranging from 1 to 7. The total score ranged from 32 to 224, with higher scores indicating better health-related QOL among patients with IBD. This questionnaire has been psychometrically validated in Chinese IBD patients (Cronbach’s *α* = 0.920) (24). In this study, the Cronbach’s *α* for the IBDQ was 0.910.

### 2.7 Statistical analysis

Statistical analyses were performed using IBM SPSS 27.0. Continuous variables were expressed as means ± standard deviations (SDs) or medians and interquartile ranges (IQRs), whereas categorical variables were reported as frequencies and percentages. Pearson’s correlation analysis was used to examine associations between normally distributed continuous variables, and Spearman’s rank correlation was used to analyze relationships between ordinal categorical variables and other variables. The sequential multiple mediation model was established using Model 6 of the SPSS PROCESS macro 4.0. Covariates included gender, disease type, disease duration, BMI, disease activity, marital status, household income, and surgical history. A bias-corrected bootstrap procedure with 5,000 resamples was used to verify the mediation effects; statistical significance was indicated when the 95% CI did not include zero. Histograms, Q–Q plots, and skewness and kurtosis coefficients were used to assess the approximate normality of continuous variables. A variance inflation factor (VIF) of <5.0 indicated the absence of multicollinearity.

To evaluate the potential impact of overadjustment, a sensitivity analysis was performed by repeating the serial mediation model (PROCESS Model 6) after removing disease activity from the covariates. The results were compared with those of the primary analysis to assess the robustness of the mediation effects.

## 3 Results

### 3.1 Sociodemographic and clinical characteristics of the research sample

A total of 327 IBD patients were enrolled in this study. Their sociodemographic and clinical characteristics are presented in Table 1. The mean age of the patients was 37.4 ± 15.3 years; 202 patients (61.8%) were diagnosed with Crohn’s disease (CD), and 125 patients (38.2%) were diagnosed with ulcerative colitis (UC). Based on the Nutritional Risk Screening 2002 (NRS2002) scores, 192 patients (58.7%) were at nutritional risk (score ≥3). The median Patient Health Questionnaire-9 (PHQ-9) score was 4.0 (IQR = 6.0), and 177 patients (54.1%) had depressive symptoms (score >4). The median FC level was 25.61 μg/g (IQR = 31.0). The mean Inflammatory Bowel Disease Questionnaire (IBDQ) score was 161.7 (SD = 24.7).

**Table 1:** Sociodemographic and clinical characteristics of 327 IBD patients.

Variable	Categories	*n* (%)	Mean ± SD/median (IQR)
Sex	Male	171 (52.3)	
	Female	156 (47.7)	
Age (years)			37.4 ± 15.3
BMI (kg/m^2^)			21.2 ± 2.9
Place of residence	Urban	192 (58.7)	
	Rural	135 (41.3)	
Disease severity	Remission	39 (11.9)	
	Mild	110 (33.6)	
	Moderate	128 (39.1)	
	Severe	50 (15.3)	
Education level	Primary or less	72 (22.0)	
	Junior high school	87 (26.6)	
	Senior high school	58 (17.7)	
	College and above	110 (33.7)	
Marital status	Unmarried	120 (36.7)	
	Married	207 (63.3)	
Employment status	Student	40 (12.2)	
	Worker	155 (47.4)	
	Unemployed	67 (20.4)	
	Retired	65 (19.9)	
Annual household income (CNY)	<30,000	109 (33.3)	
	30,000–100,000	128 (39.1)	
	>100,000	90 (27.5)	
Disease course (months)	1–12	105 (32.1)	
	13–36	142 (43.4)	
	>36	80 (24.5)	
Surgical history	No	265 (81.0)	
	Yes	62 (19.0)	
Disease type	CD	202 (61.8)	
	UC	125 (38.2)	
NRS2002 score			3.0 (3.0)
	No nutritional risk (<3)	135 (42.3)	
	Nutritional risk (≥3)	192 (58.7)	
PHQ-9 score			4.0 (6.0)
	No depressive symptoms (≤4)	150 (45.9)	
	Depressive symptoms (>4)	177 (54.1)	
FC (μg/g)			25.61 (31.0)
IBDQ score			161.7 ± 24.7

IBD, inflammatory bowel disease; CD, Crohn’s disease; UC, ulcerative colitis; BMI, body mass index; NRS2002, Nutritional Risk Screening 2002; FC, fecal calprotectin; PHQ-9, Patient Health Questionnaire-9; IBDQ, Inflammatory Bowel Disease Questionnaire; SD, standard deviation; IQR, interquartile range.

### 3.2 Correlation analysis of core variables

**Figure 2:**
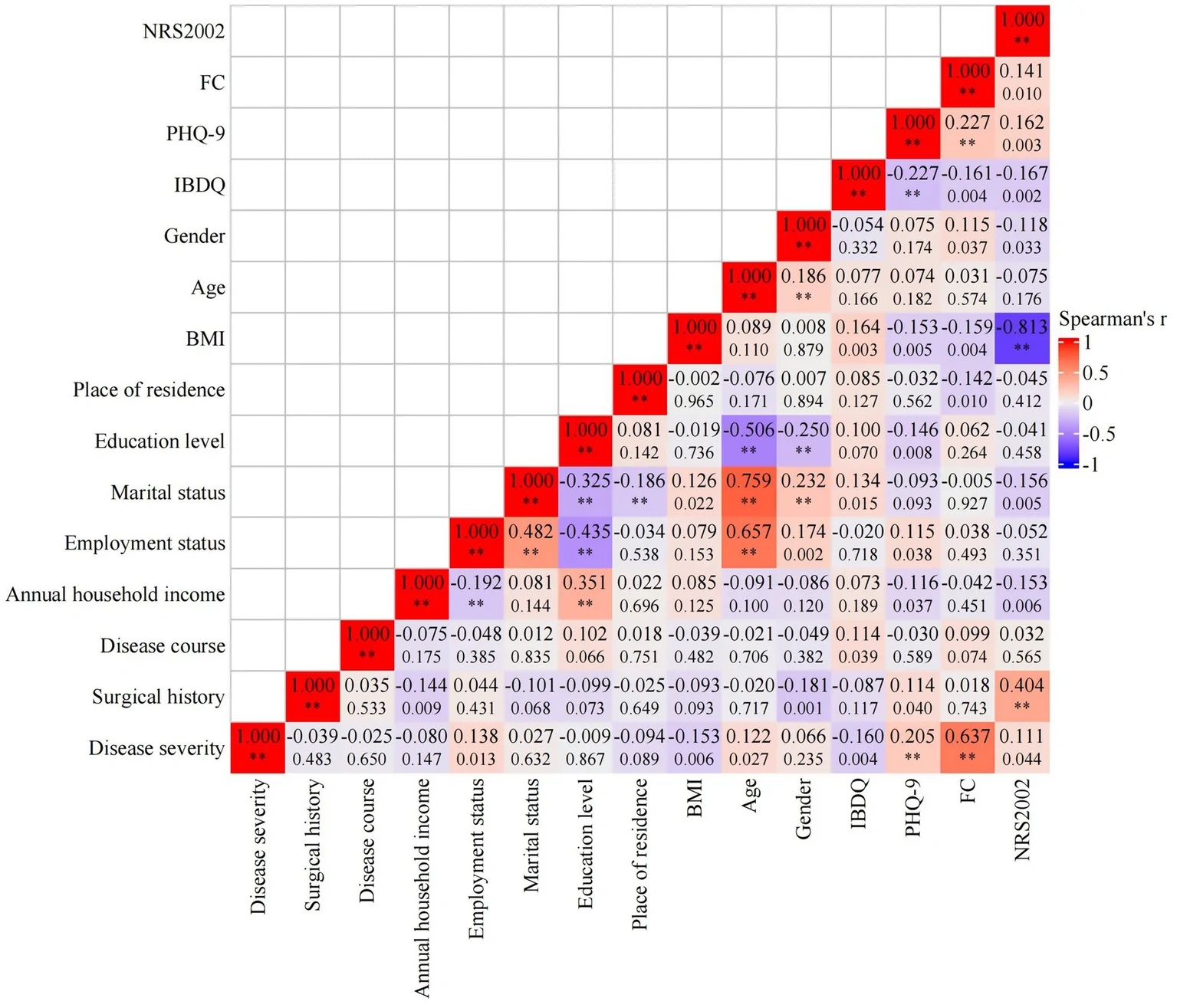
Correlations among the study variables. BMI, body mass index; NRS2002, Nutritional Risk Screening 2002; FC, fecal calprotectin; PHQ-9, Patient Health Questionnaire-9; IBDQ, Inflammatory Bowel Disease Questionnaire. **p < 0.001.

Figure 2 shows the correlation analysis among the core study variables. Nutritional risk (NRS2002 score) was significantly and positively correlated with the intestinal inflammation marker FC [*r* = 0.120, *p* < 0.05, 95% CI (0.012, 0.226)] and depressive symptoms [PHQ-9 score; *r* = 0.157, *p* < 0.01, 95% CI (0.050, 0.261)], and it was significantly and negatively correlated with QOL [IBDQ score; *r* = −0.160, *p* < 0.01, 95% CI (−0.264, −0.053)]. FC was positively correlated with depressive symptoms [*r* = 0.148, *p* < 0.01, 95% CI (0.040, 0.253)] and negatively correlated with QOL [*r* = −0.164, *p* < 0.01, 95% CI (−0.268, −0.056)]. Depressive symptoms were significantly negatively correlated with QOL [*r* = −0.249, *p* < 0.001, 95% CI (−0.348, −0.145)]. Disease activity was significantly and negatively correlated with QOL [*r* = −0.160, *p* < 0.01, 95% CI (−0.267, −0.049)]. Subgroup comparison across disease activity strata further revealed a stepwise decline in QOL with increasing disease severity: remission (173.36 ± 27.14), mild activity (164.63 ± 23.37), moderate activity (157.87 ± 23.06), and severe activity (145.24 ± 26.75). A one-way analysis of variance (ANOVA) confirmed a significant overall difference among the groups (*F* = 6.49, *p* < 0.01). Furthermore, patients with higher disease activity (assessed using the CDAI for CD and the modified Truelove and Witts criteria for UC) or a history of surgery had a higher nutritional risk. In contrast, male patients and those with a higher BMI, those who were married, or those with a higher annual household income had a relatively lower nutritional risk (all *p* < 0.05). Specifically, among 178 patients with moderate or severe disease activity, 112 (62.9%) had nutritional risk; among 62 patients with a history of surgery, 41 (66.1%) had nutritional risk.

### 3.3 Regression analysis of the mediating effects of FC and depression between nutritional risk and QOL

After controlling for covariates, including gender, disease type, disease duration, BMI, disease activity, marital status, annual household income, and surgical history, the results of the hierarchical regression analysis are presented in Table 2. When only the nutritional risk was included in the model, it showed a significant negative association with QOL (*β* = −2.596, *p* < 0.01) and a significant positive association with FC (*β* = 1.866, *p* < 0.05). After FC and depression were sequentially added to the models, FC showed a significant positive association with depression (*β* = 0.021, *p* < 0.05) and a direct negative association with QOL (*β* = −0.124, *p* < 0.05). Depression had a significant negative association with QOL (*β* = −1.417, *p* < 0.001). After both FC and depression were included in the model, the direct negative association between nutritional risk and QOL remained significant (*β* = −1.820, *p* < 0.05), suggesting that FC and depression may play important roles in the pathway linking nutritional risk to QOL. The variance inflation factors (VIFs) for all independent variables were <2.5, indicating no multicollinearity in the model.

**Table 2:** Regression analyses of the mediating effects of FC and depression on the association between nutritional risk and QOL (*N* = 327).

Regression equation		Overall fit index			Regression coefficient		
Outcome	Predictor	*R*	*R* ^2^	*F*	*β*	SE	*t*
IBDQ	NRS2002	0.160	0.026	8.581	−2.596	0.886	−2.929**
FC	NRS2002	0.120	0.014	4.759	1.866	0.855	2.182*
PHQ-9	NRS2002	0.204	0.042	7.052	0.346	0.134	2.586**
	FC				0.021	0.009	2.394*
IBDQ	NRS2002	0.301	0.091	10.759	−1.820	0.874	−2.083*
	FC				−0.124	0.056	−2.202*
	PHQ-9				−1.417	0.359	−3.949***

NRS2002, Nutritional Risk Screening 2002; FC, fecal calprotectin; PHQ-9, Patient Health Questionnaire-9; IBDQ, Inflammatory Bowel Disease Questionnaire. *p < 0.05; **p < 0.01; ***p < 0.001.

### 3.4 Chain mediating effects of FC and depression on the association between nutritional risk and QOL

**Figure 3:**
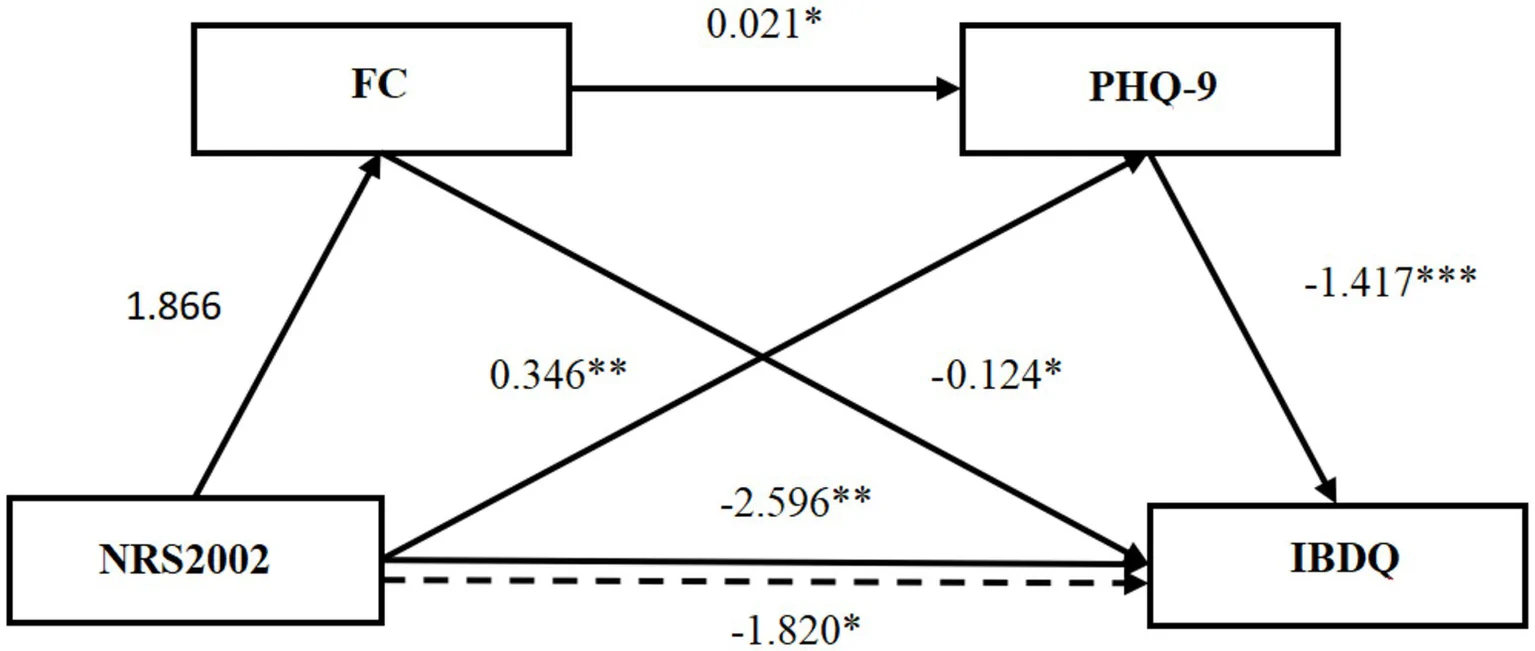
Serial mediation model of the association between nutritional risk and QOL through FC and depression. NRS2002, Nutritional Risk Screening 2002; FC, fecal calprotectin; PHQ-9, Patient Health Questionnaire-9; IBDQ, Inflammatory Bowel Disease Questionnaire; QOL, quality of life. *p < 0.05; **p < 0.01; ***p < 0.001.

The serial mediating associations were tested using the bias-corrected bootstrap method with 5,000 resamples. The model paths are shown in Figure 3, and the effect decomposition is presented in Table 3. The total association between nutritional risk and QOL was −0.456 [95% CI (−0.552, −0.360)] and was statistically significant (*p* < 0.01).

**Table 3:** Direct, indirect, and total effects of nutritional risk on QOL, with FC and depression considered as mediators.

Effects	Pathways	Standardized *β*	Boot 95%CI	Effect
Total effect		−0.456	[−0.552, −0.360]**	100.00%
Direct effect	NRS2002 → IBDQ	−0.256	[−0.364, −0.148]*	56.14%
Indirect total effect		−0.200	[−0.274, −0.126]**	43.86%
Indirect effect 1	NRS2002 → FC → IBDQ	−0.132	[−0.186, 0.078]	–
Indirect effect 2	NRS2002 → PHQ-9 → IBDQ	−0.039	[−0.074, −0.004]*	8.55%
Indirect effect 3	NRS2002 → FC → PHQ-9 → IBDQ	−0.029	[−0.051, −0.007]*	6.36%

NRS2002, Nutritional Risk Screening 2002; FC, fecal calprotectin; PHQ-9, Patient Health Questionnaire-9; IBDQ, Inflammatory Bowel Disease Questionnaire; CI, confidence interval. ^*^p < 0.05; ^**^p < 0.01.

The direct association between nutritional risk and QOL was −0.256 [95% CI (−0.364, −0.148)], accounting for 56.14% of the total effect. The total indirect effect was −0.200 [95% CI (−0.274, −0.126)], accounting for 43.86% of the total effect. The specific indirect pathways included:

① Nutritional risk → depression → QOL: This pathway had a significant mediating effect [effect = −0.039, 95% CI (−0.074, −0.004)], accounting for 8.55% of the total effect.

② Nutritional risk → FC → depression → QOL: This serial mediation pathway was significant [effect = −0.029, 95% CI (−0.051, −0.007)], accounting for 6.36% of the total effect.

③ Nutritional risk → FC → QOL: This pathway did not show a significant mediating effect [effect = −0.132, 95% CI (−0.186, 0.078)]. Although this pathway contributed numerically to the total indirect effect, its lack of statistical significance precludes meaningful interpretation.

Taken together, the total indirect effect of 43.86% comprised contributions from all three pathways. Among them, only the independent mediating effect of depression (pathway ①) and the serial mediating effect of FC and depression (pathway ②) were statistically significant, collectively explaining 14.91% of the total effect. The remaining portion of the total indirect effect (approximately 28.95%) was attributable to the non-significant pathway via FC alone. These findings indicate that the independent mediating effect of depression and the serial mediating effect of FC and depression together constitute important pathways through which nutritional risk is associated with QOL, whereas the single-mediator pathway via FC alone does not play a significant role.

### 3.5 Sensitivity analysis

To examine whether adjusting for disease activity led to overadjustment, we repeated the mediation analysis without including disease activity as a covariate. After excluding disease activity, the total effect of the NRS2002 on the IBDQ was −2.598 (*β* = −2.598, *SE* = 0.885, *t* = −2.936, *p* < 0.01). The direct effect of the NRS2002 on the IBDQ remained significant even after adjusting for FC and the PHQ-9 (*β* = −1.824, *SE* = 0.870, *t* = −2.097, *p* < 0.05). The serial mediation pathway (NRS2002 → FC → PHQ-9 → IBDQ) remained significant: FC significantly predicted the PHQ-9 scores (*β* = 0.025, *SE* = 0.009, *t* = 2.778, *p* < 0.05), and PHQ-9 significantly predicted the IBDQ scores (*β* = −1.419, *SE* = 0.356, *t* = −3.984, *p* < 0.001). The model R^2^ increased from 0.032 (NRS2002 alone predicting IBDQ) to 0.094 (full model including FC and PHQ-9). These results were substantively unchanged from those of the primary analysis, confirming the robustness of the findings.

## 4 Discussion

### 4.1 Overview of research findings and analysis of key discoveries

This study is the first to demonstrate in an Asian IBD population that FC and depression act as serial mediators of the association between nutritional risk and QOL. The serial mediation effect accounted for 6.36%, and the two variables collectively explained 43.86% of the total effect. Empirically, this result highlights the potential role of the gut–brain axis on the association between nutritional risk and QOL and offers novel targets for integrated IBD management. Notably, the direct association between nutritional risk and QOL accounted for 56.14% of the total effect, strongly suggesting that direct nutritional intervention may be the most fundamental and essential measure to optimize QOL among IBD patients. Intriguingly, the independent mediating effect of FC alone was not statistically significant. This finding implies that intestinal inflammation is indirectly rather than directly associated with patients’ QOL through psychological disorders such as depression, potentially via the gut–brain axis. This finding helps address the gap in understanding the interactive mechanism of the inflammation-psychology-nutrition cascade and provides evidence to support comprehensive intervention strategies grounded in nutritional management that simultaneously focus on inflammatory status and psychological health.

Nevertheless, the effect sizes (*R*^2^ range, 0.014–0.091) and correlation coefficients (e.g., *r* = 0.120 and *r* = 0.148) were small, warranting cautious interpretation. The clinical significance of these findings lies in the mediation pattern, namely, the identification of a sequential pathway from nutritional risk through inflammation to depression and impaired QOL, rather than in the magnitude of individual correlations or the construction of a predictive model of QOL. The identification of a statistically significant serial mediation pathway provides empirical evidence for the gut-brain axis theory, even though the path coefficients were small. The proposed integrated care model should be viewed as hypothesis-generating and requires further validation in prospective interventional trials.

### 4.2 Comparison with previous studies and mechanism explanation

The rate of nutritional risk was 58.7% among the patients enrolled in this study, representing a relatively high level in this convenience sample. Given the nonrandom sampling method, this rate should not be interpreted as the prevalence of nutritional risk in the overall IBD population. This finding was largely consistent with the latest international epidemiological data reported by the ECCO (25), further highlighting the pivotal role of standardized nutritional management throughout the course of IBD. A 2025 study by Huang et al. (26) demonstrated that intestinal inflammation can trigger endoplasmic reticulum stress in the hippocampus and consequently induce depression. Meanwhile, findings from Harvard Medical School (27) indicated that metabolites derived from gut microbiota dysbiosis can increase the risk of depression by activating systemic inflammatory responses. In the present cross-sectional study, FC levels showed a positive correlation with depressive symptoms, providing population-level evidence that supports these molecular mechanisms. This raises a critical clinical question: Could targeted nutritional support (e.g., supplementation with prebiotics, probiotics, and omega-3 fatty acids) and anti-inflammatory dietary patterns may influence these gut–brain crosstalk pathways. If they do, these interventions may improve nutritional status while simultaneously mitigating inflammation and alleviating depressive symptoms. Further in-depth exploration is recommended in future research.

Consistent with previous studies, our findings confirm that disease activity is one of the strongest factors directly affecting QOL in IBD patients (28). Active intestinal inflammation is associated with both gastrointestinal symptoms (e.g., diarrhea, abdominal pain, and hematochezia) and systemic manifestations (e.g., fatigue and weight loss), which, in turn, lead to poorer QOL (28). Meanwhile, disease activity also increases nutritional expenditure; coupled with reduced dietary intake and impaired intestinal absorption, this increases the risk of malnutrition (4). Reciprocally, malnutrition weakens immune function and impairs intestinal mucosal repair, worsening disease activity. This bidirectional vicious cycle drives the progressive deterioration of QOL in patients (4). Critically, the serial associative pathway remained statistically significant after adjusting for disease activity. This finding suggests that the pathway linking nutrition to QOL via FC and depression may operate independently of disease activity, providing empirical evidence to support integrating nutritional support therapy into the full-course management of IBD.

Regarding the apparent discrepancy between the median FC level (25.61 μg/g, below the cutoff of 50 μg/g) and the predominance of clinically active disease (88.1%), several explanations should be considered. First, FC specifically reflects mucosal inflammation, whereas clinical activity indices (CDAI and modified Truelove and Witts criteria) incorporate both objective and subjective components, such as pain, general well-being, and stool frequency. Second, normal FC levels do not entirely exclude low-grade inflammation, particularly in patients with isolated ileal CD or limited distal UC. Third, the cutoff of 50 μg/g has limited sensitivity for detecting mild endoscopic activity. Similar discordance between FC and clinical indices has been reported in previous studies (29). This discrepancy does not necessarily undermine the validity of our findings because FC remains a well-established biomarker of intestinal inflammation.

A key finding of this study was that the independent mediating effect of FC was not statistically significant, whereas the serial mediating effect of FC and depression was statistically significant. Although existing studies have shown that intestinal inflammation itself can induce intestinal symptoms such as diarrhea, abdominal pain, and hematochezia, patients’ subjective symptom perception and psychological coping mechanisms generally have a greater influence on their QOL (30, 31). The present findings suggest that the association between FC and QOL may be more pronounced when depression acts as a mediator. This may partly explain the clinical observation that some patients with high FC levels can maintain satisfactory QOL because of favorable psychological resilience. Furthermore, depression is highly prevalent among Asian IBD patients (32). Depression has been associated with aggravated intestinal inflammation through the hypothalamic–pituitary–adrenal (HPA) axis and other mechanisms, potentially forming a cycle of “inflammation → depression → aggravated inflammation” (10) and increasing the risk of disease recurrence (33). Therefore, the serial mediation model constructed in this study may reflect the role of the inflammation–depression cycle in the association between nutritional risk and QOL. Previous studies have recommended gut microbiota regulation as an adjunctive intervention for depression (34, 35), and psychological interventions have been shown to improve inflammatory levels (36). This evidence is consistent with the findings of the present study and supports an integrated intervention approach. With nutritional management as the central focus, dual strategies involving an anti-inflammatory diet and psychological support may help address the malnutrition–inflammation–depression triad and comprehensively relieve clinical symptoms.

### 4.3 Clinical significance and implications for nutritional management

The present study has several implications for the comprehensive clinical management of IBD, particularly the optimization of nutritional management: ① prioritize nutritional risk screening. Our findings support the routine use of nutritional risk screening tools such as NRS2002 for patients with IBD at initial diagnosis and follow-up, which is a fundamental step in implementing precise nutritional management; ② expand the clinical interpretation and practical application of FC. As a core biomarker of intestinal inflammatory activity, FC may also serve as a dynamic indicator of changes in intestinal inflammation associated with nutritional interventions. Serial monitoring of FC after nutritional support is initiated may enable an objective evaluation of the association between the interventions and intestinal inflammation; ③ integrate psychological assessment with nutritional management. The mediating pathway involving FC and depressive symptoms accounted for 43.86% of the total effect in this study, suggesting the limitations of interventions targeting only nutrition and inflammation. Therefore, psychological assessment using the PHQ-9 and other validated scales should be incorporated into standardized nutritional management frameworks. For patients with nutritional risk and elevated FC levels, individualized nutritional plans should be formulated together with screening for depressive symptoms. Integrated nutritional guidance combined with psychological interventions (e.g., cognitive behavioral therapy) is recommended for patients with psychological distress to improve physical and mental health simultaneously; and ④ define future research directions. Further randomized controlled trials are urgently needed to confirm the superiority of integrated interventions by comparing standard nutritional support with combined nutritional and psychobehavioral interventions in terms of FC levels, depressive symptoms, and QOL. Such evidence would provide high-level clinical support for the ability of integrated nutritional management to alleviate intestinal inflammation, relieve comorbidities, and improve the overall health of IBD populations.

Our study had several limitations. First, the cross-sectional study design cannot establish causal associations, and future cohort or interventional studies are needed for further verification. Second, all participants were recruited from four tertiary hospitals in Southwest China using convenience sampling, which may introduce selection bias and limit the generalizability of the findings. Thus, caution is required when generalizing these findings to populations in other regions or patients in primary care settings. Third, this study did not include intermediate biomarkers of the gut-brain axis, including the gut microbiota, inflammatory cytokines, and neurotransmitters, or other inflammatory markers, such as C-reactive protein (CRP) and endoscopic scores. Therefore, we could not elucidate the molecular mechanisms underlying these serial mediation effects. Future research may integrate multi-omics technologies to explore the interactive regulatory network of nutrition-microbiota-inflammation-psychology to identify additional potential targets for precise clinical intervention.

## 5 Conclusion

It should be noted that this study merely increases sensitivity and must be confirmed by further studies. Based on the biopsychosocial medical model and the gut–brain axis theory, this study is the first to demonstrate among Chinese IBD patients that nutritional risk is associated with patients’ QOL not only directly but also indirectly through the sequential pathway from FC to depression. This finding highlights two pathways through which nutritional status may influence health outcomes in IBD patients. Accordingly, clinical management may benefit from moving beyond conventional single-dimensional interventions and adopting an integrated care model centered on systematic nutritional management, with simultaneous monitoring of intestinal inflammation (FC) and screening for depression. This model provides a theoretical and empirical basis for integrated approaches aimed at alleviating inflammation, managing psychological comorbidities, and ultimately improving patients’ overall QOL.

## Data Availability

The raw data supporting the conclusions of this article will be made available by the authors, without undue reservation.
